# Psychological and Clinical Predictors of COVID-19 Severity and Outcomes

**DOI:** 10.7759/cureus.19458

**Published:** 2021-11-10

**Authors:** Mian Mufarih Shah, Sumira Abbas, Jehan Z Khan, Mehwash Iftikhar, Ayesha Jamal, Jehan Zeb Khan, Sami Ullah

**Affiliations:** 1 Department of Medicine, Medical & Teaching Institution, Hayatabad Medical Complex, Peshawar, PAK; 2 Department of Pathology, Medical & Teaching Institution, Hayatabad Medical Complex, Peshawar, PAK; 3 Department of Pharmacy, University of Peshawar, Peshawar, PAK; 4 Internal Medicine, Canadian Red Cross, Winnipeg, CAN; 5 Department of Pharmacy, Hayatabad Medical Complex, Peshawar, PAK

**Keywords:** outcomes, psychological determinants, covid severity, mental health, covid-19

## Abstract

Objective

The present study aimed to assess the psychological and clinical determinants of coronavirus disease 2019 (COVID-19) and their association with the disease severity and outcomes.

Methods

This prospective study was conducted at Hayatabad Medical Complex, Peshawar-Pakistan. Admitted patients were screened for COVID-19 with reverse transcription-polymerase chain reaction (RT-PCR) and subsequently, 250 COVID positive patients were included in the final analysis. Data were obtained from the patient's medical chart; demographic and clinical characteristics were recorded using a structured questionnaire. Psychological determinants, including anxiety and depression, were measured using the Hospital Anxiety and Depression Scale (HADS). The predictors of disease severity and outcomes (recovery vs. mortality) were also studied.

Results

A total of 250 patients were included in this study; out of which, 193 patients recovered from this deadly virus and 57 died. Based on psychological assessment, 58.4% of the enrolled COVID-19 patients had poor HADS scores. Most of the patients who died (70.2%) had severe symptoms (poor HADS scores). Similarly, 49.6% of the total cases were observed with poor HADS, and 50.9% of those who died had severe depression.

Conclusion

It is concluded from the study results that psychological distress is frequent in COVID-19 patients. Age, hypertension, fatigue, abnormal respiratory rate, oxygen saturation, ferritin, and poor HADS sore were determined as the significant predictors of COVID-19 severity and outcomes.

## Introduction

Since the coronavirus disease 2019 was found to be excessively transmissible, therefore, it was declared a pandemic and a public health emergency by the World Health Organization (WHO) due to its spread worldwide [1]. This viral infection has significantly impacted global activity; with the rise in death occurring each passing day, extensive social distancing and quarantine have been strongly recommended [2]. The psychological impacts of COVID-19 are evident, suggesting considerable vulnerability of the sufferers to stress. Social distancing, fear of being infected, and quarantine are some of the major psychological factors that are suggested to have a negative impact on the general population, causing some lasting consequences [2]. The severity of pathological and psychological symptoms among the affected patients varies considerably, from being asymptomatic to being critically ill with lethal complications [3]. Recent research in this domain suggests that several factors are responsible for such severity of the disease, including diabetes, smoking, and hypertension [4].

The rapid infectious rate, short latency, and fast recovery rates of the past viruses curtailed their long-term impact on physical and mental health and wellbeing [5]. Therefore, the well-adapted COVID-19 rapid and contagious attack on our human race has taken us by surprise [5]. Since by the current time, when this virus has infected more than millions of humans, it has equipped itself with more than twelve novel amino acid spikes, thus helping this deadly virus dig into and affect the soft tissues of the human lungs. The immune response to COVID-19 infection is complex. As the virus has the capability to mislead the body's defense system, leading to blood clots that occur due to cytokines storm, thus resulting in rupturing of the blood vessels that can lead to stroke and bleeding [6]. In addition, recent research reveals that several neurological systems of the human body get perturbed in the hospitalized infected COVID-19 patient, ranging from confusion, stroke to seizures, etc. [7].

Furthermore, long-term psychological impacts of COVID-19 are expected, and hence it is essential to investigate the clinically relevant psychological symptoms and their associated factors. According to the existing literature, it is suggested to be the major cause of anxiety and depression among the infected individuals and their loved ones [5]. Furthermore, anxiety and depression are known to alter human thoughts impacting feelings and influencing human behavior. The altered behavior due to anxiety and depression can loop back onto an individual’s thoughts; thus, this cycle gets reverberated as a spinning wheel [7], which in turn causes changes in the human neuronal circuits and connectivity of the synapse tagging the neurons of the amygdala, hippocampus, and cortex [8,9]. Among the major contributing factors of psychological distress during COVID-19 are sociodemographic, illness-related, psychosocial, and hospital-related characteristics [10,11].

However, locally in Pakistan, there is limited evidence in relation to the psychological impacts of COVID-19 so far. Herein, the present study aimed to assess the psychological and clinical determinants of COVID-19 and their association with the disease outcomes.

## Materials and methods

Study design and ethical consideration

This prospective study was conducted at Hayatabad Medical Complex, Peshawar-Pakistan, including 250 COVID-positive cases. The study was conducted according to Helsinki rules. Ethical approval was obtained from the hospital research and ethics committee (Reference no. 518/HEC/B&PSC/2021) and written informed consent was obtained from each subject before enrollment.

Inclusion and exclusion criteria

All admitted COVID-19 positive patients were included. Reverse transcriptase-polymerase chain reaction (RT-PCR) was performed for confirmation of the COVID-19 infection. While those with cognitive impairment, i.e., conditions like dementia, delirium, or any serious psychiatric condition, were excluded from the study sample.

Biochemical estimations and data collection

The data were collected from the medical charts of the 250 COVID-19 patients; socio-demographic, and illness-related clinical characteristics using a structured questionnaire. In the illness-related clinical factors, oxygen saturation, complete blood count (CBC), electrolyte profile, liver function tests (LFTs), hematological indices, and inflammatory markers (C-reactive protein [CRP], ferritin, lactate dehydrogenase [LDH], D-dimer) were included. The outcome variable psychological distress (anxiety and depression) was assessed using the Hospital Anxiety and Depression Scale (HADS) (see Appendix; Table 4), where a total score between 0 and 7 was considered normal, 8-10 score indicates borderline abnormal, and 11-21 is considered to be abnormal with severe symptoms. Furthermore, the final outcomes included the recovery and death rate among the included patients.

Statistical analysis

The collected data was entered and analyzed on Statistical Package for the Social Sciences (SPSS) version 22.0 (IBM Corp., Armonk, NY). A Chi-square test and one-way ANOVA were used to investigate the associations between potential contributing factors and outcomes (recovery or death). An additional analysis was performed to investigate the effect of psychological determinants on the outcomes (recovery vs. death) among the COVID-19 cases. Furthermore, univariate logistic regression was used to identify the predictors of mortality among the enrolled COVID-19 patients. A p-value<0.05 was considered statistically significant.

## Results

The inpatient data of 250 COVID-positive cases were included in the present study. The mean age of these patients was 54.22 ± 12.56 years; the data was stratified on the basis of hospitalization outcomes as recovery or death, as shown in Table 1. Among the clinical parameters, the blood pressure, random blood sugar (RBS), white blood cells (WBCs), platelets, ALT, oxygen saturation, ferritin, D-dimers, creatinine, prothrombin time (PT), and international normalized ratio (INR) were significantly different among the patients who recovered than those deceased (p<0.05).

**Table 1:** Baseline and clinical characteristics of the study population. Values are given as n(%) or mean±SD. *p-value < 0.05 will be considered as statistically significant Chronic obstructive pulmonary disease (COPD); hypertension (HTN); diabetes mellitus (DM); shortness of breath (SOB); temperature (Temp); hemoglobin (Hb); alanine transaminase (ALT); total bilirubin (T. Billi); alkaline phosphatase (ALP); blood urea nitrogen (BUN); sodium blood test (Na); potassium test (K); random blood sugar (RBS); hemoglobin A1c (HbA1c); erythrocyte sedimentation rate (ESR); C-reactive protein (CRP); lactate dehydrogenase (LDH); prothrombin time (PT); activated partial thromboplastin time (APTT); international normalized ratio (INR).

Variables		Overall (n=250)	Recovered (n=193)	Death (n=57)	p-value
Age (years)		54.22±12.56	51.32±12.19	64.05±8.08	0.001*
Gender	Male	165(66.0)	128(66.3)	37(64.9)	0.84
	Female	85(34.0)	65(33.7)	20(35.1)	
Comorbidities	HTN	85(34.0)	49(25.4)	36(63.2)	0.001*
	DM	82(32.8)	62(32.1)	20(35.1)	0.68
	COPD	5(2.0)	4(2.1)	1(1.8)	0.88
	Asthma	7(2.8)	6(3.1)	1(1.8)	0.59
Sign & Symptoms	Fever	224(89.6)	167(86.5)	57(100.0)	0.003*
	Cough	224(89.6)	171(88.6)	53(93.0)	0.34
	SOB	231(92.4)	177(91.7)	54(94.7)	0.45
	Fatigue	161(64.4)	112(58.0)	49(86.0)	0.001*
	Diarrhea	40(16.0)	38(19.7)	2(3.5)	0.003*
	Vomiting	48(19.2)	48(24.9)	-	0.001*
	Headache	77(30.8)	70(36.5)	7(12.3)	0.001*
Laboratory investigation	RR	25.26 ± 4.25	24.44 ± 4.28	28.05 ± 2.67	0.001*
	PR	86.52 ± 7.52	86.60 ± 8.25	86.23 ± 4.23	0.74
	Temp	100.34 ± 1.42	100.32 ± 1.51	100.44 ± 1.02	0.58
	SBP (mmHg)	135.56 ± 11.93	134.22 ± 11.56	140.09 ± 12.16	0.001*
	DBP (mmHg)	78.57 ± 8.03	79.26 ± 7.46	76.22 ± 9.42	0.01*
	Oxygen saturation	88.10 ± 5.83	89.61 ± 5.14	82.97 ± 5.09	0.001*
	RBS; 10^3^/µL	226.29 ± 118.12	196.03 ± 113.33	299.79 ± 95.73	0.001*
	WBC; 10^3^/µL	14.72 ± 5.97	14.17 ± 6.11	16.59 ± 5.06	0.007*
	Neutrophils; 10^3^/µL	83.64 ± 8.65	83.45 ± 9.54	84.29 ± 4.50	0.52
	Lymphocytes; 10^3^/µL	11.13 ± 6.70	10.77 ± 7.30	12.36 ± 3.91	0.12
	Monocytes; 10^3^/µL	3.77 ± 1.93	3.83 ± 2.01	3.56 ± 1.63	0.35
	Platelets; 10^3^/µL	265.45 ± 92.28	258.95 ± 93.72	287.47 ± 84.28	0.04*
	Hb; g/dL	12.66 ± 1.87	12.54 ± 2.00	13.05 ± 1.23	0.07
	ALT; IU/L	41.80 ± 17.81	43. 24 ± 18.99	36.95 ± 11.97	0.02*
	T.BILLI; µmol/L	0.61 ± 0.25	0.60 ± 0.26	0.65 ± 0.21	0.18
	ALP; IU/L	98.64 ± 36.78	99.18 ± 40.00	96.82 ± 22.92	0.67
	BUN; mmol/L	43.90 ± 20.76	45.18 ± 21.48	39.56 ± 17.59	0.07
	Creatinine; mg/Dl	0.94 ± 0.38	0.98 ± 0.38	0.80 ± 0.34	0.002*
	Na; mmol/L	136.60 ± 6.45	136.62 ± 6.67	136.54 ± 5.72	0.94
	K; mmol/L	4.30 ± 0.66	4.31 ± 0.68	4.27 ± 0.60	0.69
	HbA1c (%)	6.38 ± 1.42	6.32 ± 1.44	7.07 ± 1.04	0.39
	ESR; mm/hr	33.68 ± 19.05	34.37 ± 22.15	32.26 ± 9.94	0.53
	CRP; mg/L	8.24 ± 6.42	7.89 ± 6.76	9.43 ± 4.98	0.11
	LDH; U/L	554.89 ± 199.91	541.17 ± 209.55	601.37 ± 155.92	0.05
	Ferritin level; ng/ml	1090.98 ± 583.21	909.53 ± 493.62	1705.34 ± 423.45	0.001*
	D-dimer; ng/ml	3.90 ± 2.92	3.36 ± 3.03	5.71 ± 1.54	0.001*
	PT	13.26 ± 2.84	13.49 ± 3.15	12.49 ± 1.10	0.02*
	APTT	28.80 ± 4.48	29.02 ± 5.09	28.07 ± 0.53	0.16
	INR	1.14 ± 0.41	1.17 ± 0.49	1.04 ± 0.18	0.04*

With respect to psychological assessment, 58.4% of COVID patients had abnormal HADS scores, i.e., severe psychological distress. Most of the patients who died (70.2%) had severe symptoms (abnormal HADS scores). Similarly, 49.6% of the total cases were observed with abnormal HADS, and 50.9% of those who died had severe depression, as shown in Table 2. 

**Table 2:** Anxiety and depression scores of the study population with respect to outcome. Values are given as n(%). *p-value < 0.05 will be considered as significant.

Variables		Outcome			p-value
		Overall (n=250)	Recovered (n=193)	Death (n=57)	
Anxiety	Normal	51(20.4)	47(24.4)	4(7.0)	0.02*
	Borderline abnormal	53(21.2)	40(20.7)	13(22.8)	
	Abnormal	146(58.4)	106(54.9)	40(70.2)	
Depression	Normal	31(12.4)	26(13.5)	5(8.8)	0.63
	Borderline Abnormal	95(38)	72(37.3)	23(40.4)	
	Abnormal	124(49.6)	95(49.2)	29(50.9)	

Several factors were associated with COVID-19 severity and outcomes (recovery and mortality) as per the univariate analysis. Age, HTN, fatigue, RR, SBP, DBP, oxygen saturation, RBS, ferritin, and HADS (A) were defined as the significant predictors of mortality among the enrolled COVID-19 patients (p<0.01), as shown in Table 3.

**Table 3:** Predictors of COVID-19 severity and outcomes (recovery/mortality). *p-value < 0.05 will be considered as significant Hypertension (HTN); absolute lymphocytic count (ALC); neutrophil-to-lymphocyte ratio (NLR); lactate dehydrogenase (LDH); body mass index (BMI); random blood sugar (RBS); white blood cell (WBC); alanine transaminase (ALT); systolic blood pressure (SBP); diastolic blood pressure (DBP); prothrombin time (PT); international normalized ratio (INR); alanine transaminase (ALT); Hospital Anxiety and Depression Scale (HADS).

Variables	OR (95% CI)	p-value
Age	1.105(1.070-1.141)	<0.001*
HTN	5.038(2.688-9.443)	<0.001*
Fatigue	7.729(3.285-18.186)	<0.001*
Diarrhea	0.406(0.073-2.241)	0.301
Headache	0.269(0.105-0.694)	0.007
RR	1.363(1.184-1.567)	<0.001*
SBP	1.086(1.038-1.135)	<0.001*
DBP	0.911(0.863-0.962)	<0.001*
Oxygen saturation	0.795(0.734-0.861)	<0.001*
WBC	1.098(0.996-1.210)	0.059
Platelets	1.003(0.997-1.009)	0.397
ALT	0.960(0.922-1.000)	0.047
Creatinine	0.360(0.082-1.582)	0.176
RBS	1.006(1.002-1.011)	0.004*
Ferritin	1.003(1.002-1.004)	<0.001*
D-dimer	1.169(0.975-1.402)	0.091
PT	0.867(0.609-1.235)	0.429
INR	0.429(0.076-2.414)	0.337
HADS	1.788(1.160-2.757)	0.009*

## Discussion

This study was aimed to assess the prevalence of psychological distress among COVID-19 and its association with disease outcomes in terms of recovery and death. A considerable rate of psychological distress (anxiety and depression) was observed among the enrolled COVID-19 patients in the present study. Most of the patients who died had high anxiety and depression scores, indicating increased psychological distress among most severe COVID-19 cases. Our findings are in line with the existing literature [12-14].

Zhang et al. reported mild to moderate symptoms of anxiety and depression among 21% and 29% of patients, respectively [13]. This is also supported by a similar observational study; Bo and colleagues reported symptoms of PTSD in more than 90% of the hospitalized COVID-19 patients [14]. Symptoms of anxiety may appear minor in the beginning, but they could quickly intensify over the course of a few days [15]. Because of psychological symptoms severity in isolated and hospitalized COVID-19 patients, it appears that using psychological and psychiatric counseling methods, whether in person or through online hospital systems, can be an effective way to manage patients' clinical conditions [16].

Approximately 14.3% of the deaths are attributed to mental disorders globally. Hence it is recognized as the considerable cause of morbidity and mortality [17]. The association between mental health and COVID-19 is bi-directional, although both the facts are well-known and widely studied. That is, there is an increased risk of SARS-CoV-2 infection among patients with mental disorders [18]. Secondly, the COVID-19 patients with no previous history of psychological distress develop severe anxiety and depression, which may be due to prolonged quarantine, insufficient information, isolation, boredom, and social stigma, etc. [18,19].

Other than the psychological determinants, the patient's symptomatology, clinical profile, and management protocol were also followed. The fever appeared to be the most frequent symptom in the COVID-19 cases. Fever, cough, and fatigue appear to be the predominant symptoms among participants in several studies conducted to assess the risk factors for severe COVID-19, with fever being the most prevalent initial symptom [20,21]. Furthermore, among the laboratory parameters, oxygen saturation, ferritin, D-dimers, creatinine, PT, and INR were significantly altered, and with that, these estimations greatly varied among recovered and deceased patients (p<0.05). These findings are also supported by similar local studies [22,23].

Among the significant predictors of COVID-19 severity and outcomes were age, hypertension, fatigue, abnormal respiratory rate, oxygen saturation, blood glucose level, ferritin, and HADS. Consistent with this, a local case series from Pakistan identified age, presence of comorbidity, high neutrophil-to-lymphocyte ratio (NLR), LDH, and CRP levels as strong predictors of mortality among COVID-19 patients [22], which is also supported by other international studies [24]. But to the best of our knowledge, none of the studies focused on the psychological determinants as the potential predictors of mortality among COVID-19 patients, specifically in Pakistan. 

Although the present study provides significant insight on the prevalence of psychological distress among COVID-19 patients, there are certain limitations that must be recognized. The present study did not identify the predictors or contributors of psychological distress among the COVID-19 patients. Furthermore, the sample size was small and specific to a single-center only.

## Conclusions

A considerable proportion of the enrolled COVID-19 patients displayed psychological distress (anxiety and depression). Furthermore, the mortality risk accelerated with increasing age, the incidence of hypertension, fatigue, decreased oxygen saturation, increased ferritin levels, and high HADS score. Therefore, these are determined as the potential predictors of COVID severity and outcomes (recovery vs. mortality).

## Appendices

**Table 4:** Hospital Anxiety and Depression Scale (HADS). Tick the box beside the reply that is closest to how you have been feeling in the past week. Please check you have answered all the questions Scoring: Total score: Depression (D) 0-7 = Normal 8-10 = Borderline Abnormal (borderline case) 11-21 = Abnormal (case)

D	A		D	A	
		I feel tense or 'wound up':			I feel as if I am slowed down:
	3	Most of the time	3		Nearly all the time
	2	A lot of the time	2		Very often
	1	From time to time, occasionally	1		Sometimes
	0	Not at all	0		Not at all
		I still enjoy the things I used to enjoy:			I get a sort of frightened feeling like 'butterflies' in the stomach:
0		Definitely as much		0	Not at all
1		Not quite so much		1	Occasionally
2		Only a little		2	Quite Often
3		Hardly at all		3	Very Often
		I get a sort of frightened feeling as if something awful is about to happen:			I have lost interest in my appearance:
	3	Very definitely and quite badly	3		Definitely
	2	Yes, but not too badly	2		I don't take as much care as I should
	1	A little, but it doesn't worry me	1		I may not take quite as much care
	0	Not at all	0		I take just as much care as ever
		I can laugh and see the funny side of things:			I feel restless as I have to be on the move:
0		As much as I always could		3	Very much indeed
1		Not quite so much now		2	Quite a lot
2		Definitely not so much now		1	Not very much
3		Not at all		0	Not at all
		Worrying thoughts go through my mind:			I look forward with enjoyment to things:
	3	A great deal of the time	0		As much as I ever did
	2	A lot of the time	1		Rather less than I used to
	1	From time to time, but not too often	2		Definitely less than I used to
	0	Only occasionally	3		Hardly at all
		I feel cheerful:			I get sudden feelings of panic:
3		Not at all		3	Very often indeed
2		Not often		2	Quite often
1		Sometimes		1	Not very often
0		Most of the time		0	Not at all
		I can sit at ease and feel relaxed:			I can enjoy a good book or radio or TV program:
	0	Definitely	0		Often
	1	Usually	1		Sometimes
	2	Not Often	2		Not often
	3	Not at all	3		Very seldom
